# Cities and floods: A pragmatic insight into the determinants of households’ coping strategies to floods in informal Accra, Ghana

**DOI:** 10.4102/jamba.v11i1.608

**Published:** 2019-01-10

**Authors:** Kwaku Owusu Twum, Mohammed Abubakari

**Affiliations:** 1 Department of Planning, Kwame Nkrumah University of Science and Technology, Ghana; 2 Rural Environmental Care Association, Accra, Ghana

## Abstract

Floods are common events that confront many cities in the developing world. Ghana, a developing country, is persistently challenged with flood events, especially in its major cities. In informal Accra, for instance, despite the severity of flood effects and its associated threats, poor informal residents continue to stay. As a result, these poor urban dwellers have developed local coping strategies made up of mitigation and reactive measures to manage and adapt to flood hazards through their preceding experiences. In this article, we have embraced the convergent parallel mixed method of case study design to echo and explore (1) the major effects of preceding floods on informal households, (2) the local informal coping strategies adopted by households to mitigate and respond to flooding and its effects in the future and (3) the determinants of the coping strategies of households that underpin their continual stay in spite of flood risks in Alajo, an urbanised suburb in Accra metropolis noted as one of the slum communities that easily flood in Ghana. Our analysis has used a mix of qualitative and quantitative data collected from both secondary and primary sources as well as a conceptualised model known as disaster resilience of place. The key findings (Alajo has low degree of adaptive resilience to major floods which might occur in the future because of the lack of social learning in the coping strategies developed through several years of lessons learnt from perennial floods) and proposals (local coordination in implementing the coping strategies to flooding, state support of the local strategies and adoption of rainwater harvesting) also make contributions to managing urban floods in informal settlements in the developing world.

## Introduction

Over the past few decades, urban communities have become the central hub for human existence. People prefer to live in urban areas instead of rural communities. An estimation made by the UN-Habitat (2010) suggests that about 70% of the world’s population will live in urban centres by 2050. Even before 2050, the world should expect more than 60% of its population living in urban areas by 2030, with Africa recording a rapid rate of urbanisation (Adegun 2011; UNCHS 2007). The rising spate of urban share of population has led to the development and continual expansion of informal settlements and slums (UN-Habitat 2010), which are susceptible to disasters such as flooding (De Risi 2003).

The frequency and severity of flooding in African cities have been heightened (Douglas et al. 2008) ranking second to Asia (Tschakert et al. 2010) which has the highest rate of urbanisation in the world. In Ghana, for every ten people, more than four live in the urban centres (Songsore 2009), with over half (58%) of the urban population living in slums (as indicated in Growth and Poverty Reduction Strategy, 2006–2009). The slum dwellers are persistently confronted with floods, especially in Accra, where more than ten floods were recorded between 1995 and 2007 characterised by deaths, household displacements and huge loss of infrastructure, properties and capital (Aboagye 2012). Rapid urbanisation, going hand in hand with increasing concentration of human activities, densities and congestion, pollution and impermeable surfaces without commensurate measures have increased exposure and vulnerability to flooding triggered by heavy rainfall, and these have been common contributors to flood events in Africa, including Ghana (Douglas et al. 2008). Ghana’s urbanisation is widely disorganised, and as such the millions of desperate and optimistic rural migrants who enter the cities have no appropriate place to stay and/or work. In order to fit into the cities, these groups resort to informal locations, usually flood-prone areas that constantly put their lives and properties under serious threat (Douglas et al. 2008; Jha, Bloch & Lamond 2012; Satterthwaite 2007). The frequency of floods in informal areas in Ghana is enormous, with severe negative implications on the poor and marginalised people *vis-à-vis* individuals living in formal locations (Owusu & Afutu-Kotey 2010). The informal areas are inundated centres that are industrially ‘rejected’, and are characterised by unsecured slopes, high levels of pollution and hazards (Satterthwaite et al. 2011). These are significant features that have made the economic values of informal areas considerably low, thus serving as attraction points and preferred destinations for the urban poor amidst the dangers posed for staying there. These poor urbanites are more concerned about the economic gains rather than the threats and risks they are exposed to. As such, they live as ‘survivors’ under the mercy of flood disasters, where their lives and properties are continuously threatened. Although this may seem irrational, the informal population continue to rise and their influence consolidates progressively. In Kumasi, the second major city in Ghana, Adjei-Mensah et al. (2013) revealed strikingly that more than 90% of informal dwellers had neither thought of moving away from their informal settlements (91.6%) nor made plans to live in formal settlements (93.7%). This revelation is quite surprising, but these people live in such areas as they have no option because of their limited resources, and as such they are forced to live and perceive such areas as ‘right place of abode’ for their existence.

In managing floods in Ghana, city authorities have commonly embraced the issuance of eviction notices and various evacuation threats to informal residents (Owusu & Afutu-Kotey 2010), an approach that has proven unsuccessful but is constantly adopted as if there are no alternative ways of controlling urban informality. In the phase of floods, local authorities tend to give more priority to commercial and administrative areas, whereas the poor informal communities, which are the most affected centres, are less prioritised (Douglas et al. 2008). This has therefore induced informal dwellers living in flood-prone areas to develop local mitigation measures and reactive responses to manage and adapt to flood disasters (Amoako 2014). These informal dwellers are able to sustain flood events to some degree despite the severity of its impacts, which has caused them to be adamant to the threat of city authorities. These local mitigation measures and responses have been shaped from the incremental learning and the determinant factors including topography, locational disadvantage and the absence of state support. This research has used Alajo, an informal settlement in the Accra metropolis of Ghana, as a case study. Firstly, it has examined the preceding effects of flooding on the informal households in the community. This is followed by the exploration of how these households prepare and respond to flooding, and what have influenced these measures used to mitigate and respond to the flooding. The findings have been discussed conceptually using the disaster resilience of place (DROP), a model developed by Cutter et al. (2008) for place-specific disaster studies.

## Understanding urban informality and flood, flood vulnerability, flood hazards and flood copingstrategies for local flood management

### Urban informality and floods: An overview

Urban informality is a popularly discussed issue in urban literature. It is thus predominantly known and persistently explored by urban planners and development partners. Scholarships on urban informality continue to evolve as an attempt to unravel its complexity and to find responsive measures to appropriate it into urban development interventions. De Soto (2000) has extensively studied urban informality through an economic lens. He subsequently defined informal economy as ‘people’s spontaneous and creative response to the state’s incapacity to satisfy the basic needs of the impoverished masses*’* (as in The Other Path 1989:14). Subsequently, through continuous studies of informality, he presented it as ‘heroic entrepreneurship’, where the poor urbanites earn their source of income for existence. While Hall and Pfeiffer (2000) generally perceive urban informality as an urban development problem, Roy and Al-Sayyad (2004) have argued that informality in itself should not outrightly be tagged as a problem, rather a process that governs how urban communities are transformed through a series of transactions that connect formal and informal spaces and economies to one another through a system of norms and logic. In the phase of disparity in understanding and presenting urban informality, characterised by the lack of consistency in both theoretical and empirical research (Guha-Khasnobis, Kanbur & Ostrom 2006), the choice of how to depict urban informality should be influenced by the context of its discussions (Heintz 2012). Following discussions, urban informality can generally be divided into two components: informal settlements and informal economic sector (Porter et al. 2011). Thus, the lack of consensus is deepened by the angle in which scholars tend to view the term. In this article, we view urban informality in a similar lens as Duminy (2011), who presented it as a series of behaviours and practices evolving within cities, which are relatively unregulated or uncontrolled by the state or formal institutions. Informality is widely varied in terms of housing and jobs, and it could offer an avenue not only for the informal dwellers, but also for the formal residents in the urban centres (Werna 2001). Thus, informality is not merely composed of socio-spatially marginalised people, but also well-to-do individuals who work within the formal sector and/or individuals who live in formal communities but work within the informal sector.

The UN-Habitat (2003) aligns informality to informal settlements by clearly indicating that informality is closely associated with issues of illegal occupations on lands and unauthorised houses and structures, a phenomenon very common in the developing world. This is explained by the fact that emerged or emerging physical developments do not meet planning approval and structures are not in conformity with building and zoning regulations (Fekade 2000). Because of the nature of informality in the developing world, Elgin and Oztunali (2012) have contended that urban informality is rife and has serious social, political and economic development challenges. The rise in disasters, notably, flooding, and the spread of overcrowding, congestion, crime and theft have direct connection with urban informality (Twumasi-Ankrah 1995). Paying attention to informality and floods, Dodman, Bicknell and Satterthwaite (2012) agree that informality contributes to flood events. This comes about when a high proportion of lower income groups settle on hazardous sites (sites at risk of floods or landslides), which they do so for the lack of safer lands which meet their economic standards of affordability. The dwellers of such areas are vulnerable to floods and other hazards because of their huge concentration and densification of businesses within the same locations (Douglas et al. 2008). This is further worsened by the poor road access to informal communities which serve as a great obstacle for easy exit during emergencies. The increase in concentration leads to the expansion of informal areas into nearby formal communities and occupation of marginal lands on the urban periphery (Satterthwaite et al. 2011). This is incrementally manifested through the alteration of natural landscapes, land uses and land cover, which add to flood hazard problems. This is because, apart from the alteration of natural landscapes, land uses and land cover, informal practices such as insufficient removal of refuse and drainage infrastructure for available population are transcended to such nearby formal areas exposing such areas to flood risks. Sakijege, Lupala and Sheuya (2012) expatiate on the contribution of informality to flood events by indicating that as densification increases, the run-off water from the roofs of buildings alters the urban land cover and land surface, and this is further exacerbated by poor solid waste management practices of informal residents. Thus, floods are caused by not only natural circumstances but also human activities (Douglas et al. 2008). As climate change influences flood events (Amoako 2014), so does local urban change increase the likelihood of floods because of the adjustments to the urban land surface and water passageways as a result of human activities such as structural developments, flooring or paving (impervious surfaces), soil compaction, the elimination of vegetation and the digression of natural flows. Subsequently, these informal areas play a major role in the cause and severity of flood effects.

### Flood vulnerability, flood hazard and coping strategies for building community-level resilience to floods

#### Flood vulnerability and flood hazard

To understand flood vulnerability, the term ‘vulnerability’ needs to be explored and appreciated. Although the origin of the term can be traced from the field of geography and natural hazards, many other related disciplines now use the term in a contextualised manner (Gow 2005; O’Brien et al. 2004a). Others relate the term to concepts such as marginality, susceptibility, resilience, fragility and risk (Liverman 1990), as such making it complicated to define. Hence, Kasperson et al. (2005) concluded that a single accepted definition for vulnerability is non-existence. Brooks et al. (2005) have also reiterated that one can meaningfully define vulnerability based on specific hazard(s) of a particular system so as to differentiate between current and future vulnerability. On the basis of the ambiguity in meanings of vulnerability, we contextualise our definition of the term in tandem with flood events in informal urban communities. We adapt Turner II et al.’s (2003) general definition of vulnerability as ‘the degree to which a system is likely to experience harm due to exposure to a hazard’ and contextualise it *vis-à-vis* urban informality and floods. Flood vulnerability is thus defined as the degree to which informal urban communities are likely to experience harm because of their exposure to flood hazards. We further define flood hazards based on the adapted definition of hazard by the United Nations (2004) as ‘a potentially damaging physical event, phenomenon or human activity that may cause the loss of life or degradation’. Based on this premise, flood hazard is defined in this article as potentially damaging flood event induced by nature or human activities that may cause the loss of life or injury, property damage, social and economic disruption or environmental degradation in informal urban settlements.

#### Flood coping strategies: An approach to building local adaptation and strengthening resilience to flood hazards

Before we look into the specific coping strategies of informal communities to flood hazards, it is prudent to look into the terms ‘coping’, ‘adaptation’ and ‘resilience’ and establish clearly what they stand for in this research.

Coping is made up of immediate and short-term measures to an event, culminating ‘here and now’ capacity of a system and/or community to mitigate and respond to the event (Birkmann 2011). Adaptation is somewhat a long-term process that entails a systematic approach of learning (either planned or spontaneous in nature), experimentation and change before or after a disaster (Yohe & Tol 2002; see also Pelling 2010). Resilience is the capacity of a system and/or community to prevent, mitigate and/or cope with risk, and recover from shocks (FAO 2012). Resilience is a state informed by sound coping measures and adaptation, and it takes considerable period of time for a community to reach. Carpenter et al. (2001) have revealed that resilience can be specified in the context of vulnerability as ‘resilience of what to what’. A system could thus be said to be resilient when it is less vulnerable to shocks across time and can recover from them. Communities can build resilience when they adapt to risk through incremental and social learning from adaptation measures.

**FIGURE 1:**
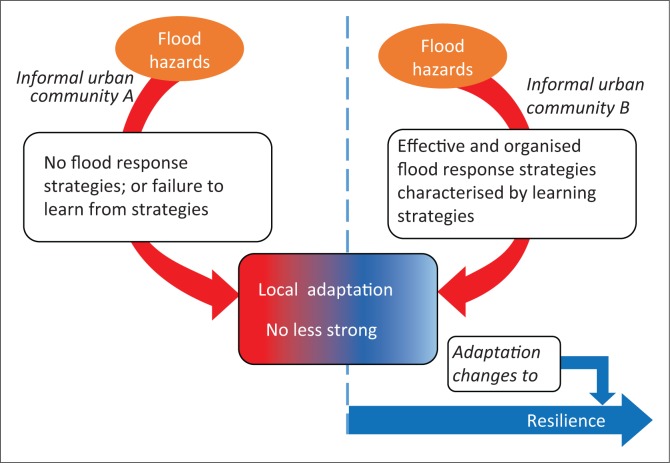
Local flood adaptation and resilience.

In the context of urban informality and flooding, we can infer that communities can systematically learn from their flood coping strategies (short-term), which can inform their adaptation measures to flood hazards. Through this, informal communities can subsequently strengthen their adaptation to floods, helping them to reach a state of resilience to flood hazards over time (see Figure 1). Placing emphasis on coping strategies of informal communities to floods, Douglas et al. (2008) have indicated that the strategies are mostly individually initiated and disorganised, hence making their contributions to building local adaptation, and subsequently helping communities to reach a state of resilience to floods questionable. These coping strategies could entail the construction of barriers to impede the inflow of water into houses during flood hazards, building temporal shelters (Adelekan 2010), moving important items to safe places in the event of flooding, creating high places in homes using furniture, stones and blocks where items can be kept temporarily during floods and treating drinking water through boiling to reduce the risk of water-related diseases (Douglas et al. 2008). In a related study conducted by Sakijege, Lupala and Sheuya (2012), flood coping strategies at the household level were the use of sandbags and tree logs, water boiling and chemical treatment, raised doorsteps and pit latrines, construction of proactive walls and elevation of house foundations, temporal movement to safe places and provision of pipe outlets to drain off water during heavy rainfalls. Sakijege, Lupala and Sheuya (2012) further indicated that some community-based interventions such as control of housing development, protest, attempts to request companies that have contributed to flooding to compensate community members and initiation of communal solid waste management practices have been implemented but were unsuccessful. In light of the difficulties faced by informal residents in organising themselves in implementing flood coping strategies for the entire community, Amoako (2012) has revealed that individual- and household-based interventions have produced very little success in local flood adaptation, helping them to incrementally build resilience to flood hazards. Informal community members are somehow able to cope with flood events in the short-term, but still have high risk to flood hazards despite flood coping strategies. This is because of their exposure to floods, as they still remain in areas where flooding occurs. Perhaps, the uncoordinated nature of local flood coping strategies among the informal settlers is a reason for their less recognition and support by formal state institutions.

Figure 1 shows two informal urban communities, A and B, with B having built resilience to flood hazards through strong adaptation ensured by the effective and organised flood coping and/or response measures characterised by learning strategies by the local dwellers. On the other hand, community A has no or less adaptation because of either the lack of local flood coping and/or response measures to adapt to flooding or community members have failed to incrementally learn from the measures put in place to inform adaptation helping them to build resilience over time.

## Disaster resilience of place: Adopted model for understanding informal households’ responses to floods

This research adopted the DROP model developed by Cutter et al. (2008) as a place-based model for understanding how a community develops resilience to natural disasters such as floods through adaptation. The DROP model explains, in light of theory, how local communities build resilience to disasters through incremental learning, and practical mechanisms to which real-life disaster problems can be addressed in real places. The model is underpinned by three critical assumptions which have justified why the researchers adopted it as a conceptual model for this research:

- The model can be used to study a wide range of disaster problems including floods, a perennial disaster in which this article tends to explore in informal Accra.
- The model’s applicability is at the local level instead of national and/or international level, hence making it appropriate for adoption.
- The model focuses on the connection between social systems, natural systems and physical or built environment and how they contribute to, and can be used to manage place-specific disasters such as floods.

**FIGURE 2:**
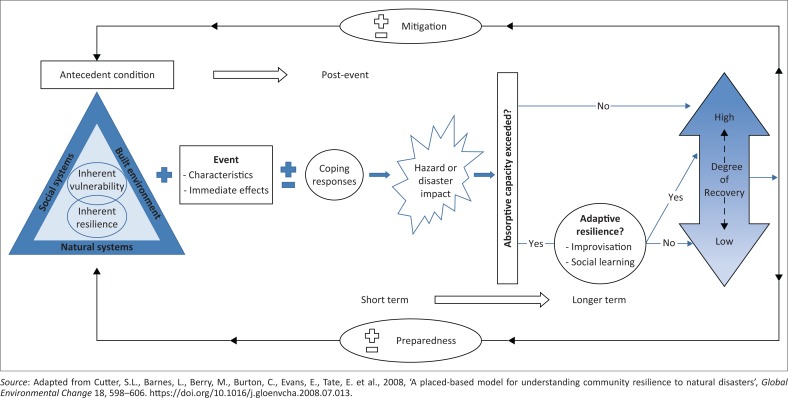
The disaster resilience of place model.

Place-specific factors between social systems, natural systems and the physical or built environment influence the degree of inherent vulnerability and inherent resilience (see nested triangle in Figure 2) of a particular community to disaster. These place-specific factors vary and could entail poverty, rapid population growth, disorganised buildings because of poor governance, socio-economic marginalisation (Braun & Aßheue 2011) and features of the landscape that increase exposure to hazards. The inherent process both in the context of vulnerability and resilience happens at the local scale and is influenced by the multi-scale factors interconnected by social systems, natural systems and the built environment (as in the bigger triangle). Cutter et al. (2008) treated the factors as ‘antecedent conditions’ which interact with the disaster event characteristics. Disasters then occur when people exposed to the hazards are vulnerable to their effects. The disaster event characteristics include the duration of the disaster, its number of occurrences (frequency), intensity, magnitude and rate of onset. These immediate effects are either reduced through effective coping strategies (indicated with a minus [−] sign) or intensified through the absence of any strategy (indicated with a plus [+] sign). Thus, using informal communities as an example, households’ use of local coping strategies to disaster adaptation such as floods could help reduce the immediate effects of the flooding. The strategies in the context of this study have been espoused in the findings section of the article.

The disaster impacts are a combination of antecedent conditions, disaster event characteristics and coping strategies embraced by local communities. The use of predetermined coping strategies by local communities in order to withstand the impacts of the occurrence of a known event or disaster underpins the absorptive capacity of such communities. Absorptive capacity is the ability of a system (a community and/or households) to survive shocks and stress – that is, the system keeps functioning in the phase of disaster (Boubacar et al. 2017). Communities with effective implementation of appropriate coping measures will undeniably be able to reduce the impacts of an event or disaster should it occur and also their absorptive capacity to the event or disaster will not be exceeded. At this point, the degree of recovery of the affected community will be high. On the other hand, the absorptive capacity of local communities to an event or disaster could either be exceeded when coping strategies are not sufficient to withstand the impacts of the event or disaster should it occur or the occurrence of the event or disaster will have severe impacts which exceed the coping strategies of local communities to the event or disaster. For instance, if households’ coping strategies to flood events which occur perennially are not adequate or the occurrence of the flood events is severe such that its impacts will absorb the local coping strategies of the households, then the absorptive capacity of the community will be exceeded. At this point, the community could, perhaps, exercise adaptive resilience through immediate actions during the disaster and social learning to help in the recovery process after the disaster. Adger et al. (2005) have unravelled that social learning involves differences in adaptations as well as embracing and building strong social integration at the local level for group-based actions to recover from the occurrence of the disaster. The social learning becomes a process and feeds into future coping strategies for pre-event preparedness advancement, which subsequently strengthens the absorptive capacity of communities for future events through inherent resilience indicated by the feedback loops in Figure 2. It is worthy to note that once coping strategies are ineffective and local absorptive capacity is exceeded, households can learn some lessons (*lessons learnt*) in terms of what they did right and wrong, and this becomes future recommendations which could either be implemented or never implemented by individual households. This makes ‘lessons learnt’ quite apart from social learning which considers collective actions to deal with a disaster, which also feed into future strategies of households. Through social learning (collective initiative based on shared experiences and resources), with or without implementation of lessons learnt (informed by individuals’ experiences and means to deal with a disaster), households are able to build adaptive resilience. Once adaptive resilience is built, households can successfully cope with risk and recover from shocks associated with disasters. The degree of recovery for such households becomes very high. Alternatively, a low degree of recovery occurs when after absorptive capacity has been exceeded, ‘NO’ (as indicated in Figure 2) adaptive resilience is built by the community. This occurs when coping strategies are either in non-existence or are very poor. Building resilience is very essential as new knowledge is gained through the process which provides feedbacks to modify the new coping strategies. The existence of social learning and implementation of lessons learnt in the entire process provide a potential platform to enhance preparedness (+) and mitigation (+); otherwise, preparedness and mitigation will be affected negatively (-). The DROP model provides useful insights into community-based disaster studies, but one limitation we identified is the failure of proponents to admit that ‘external supports’ can help in recovery process of communities whose absorptive capacity is exceeded as a result of ineffective and unsound coping strategies. The model thus attempts to outline how vulnerable communities can become resilience over time through local measures without due recognition of *‘external supports*’, especially from responsible state officials. The DROP model must pay credence to the development of guidelines that can recognise structural, economic, social and environmental policy changes as indicated by Cutter et al. (2008) who developed the model.

## Study context and methods

### Study context

**FIGURE 3:**
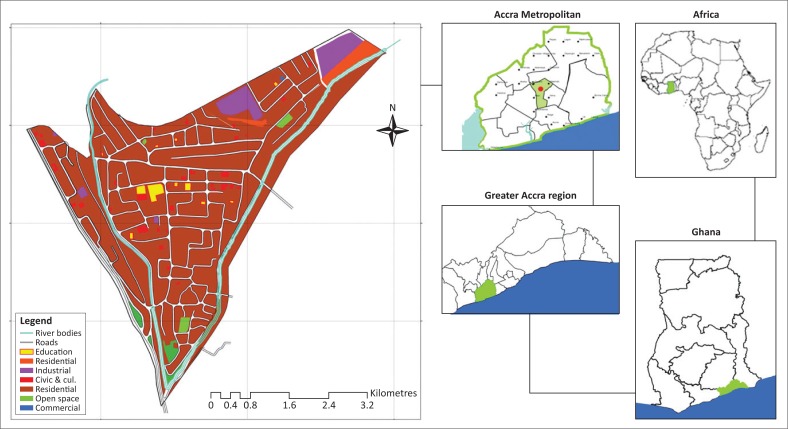
Map of Alajo showing the various land uses.

**FIGURE 4:**
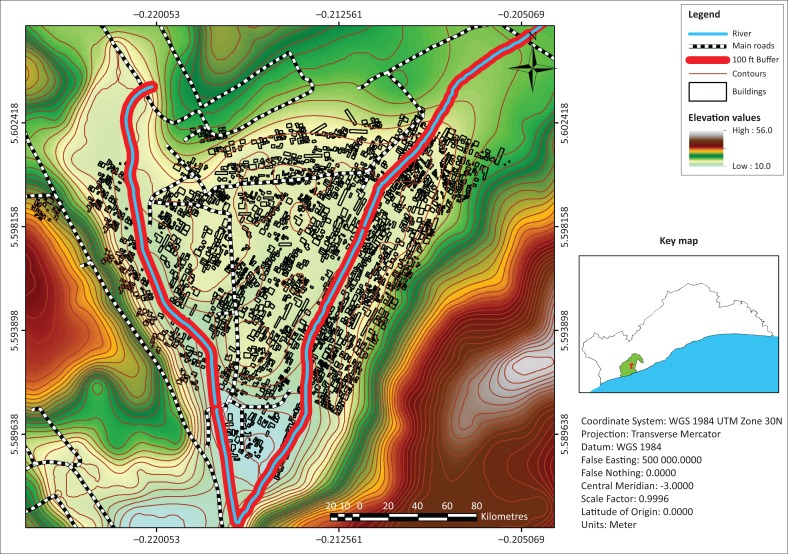
Map of Alajo showing areas of flood exposure.

**FIGURE 5:**
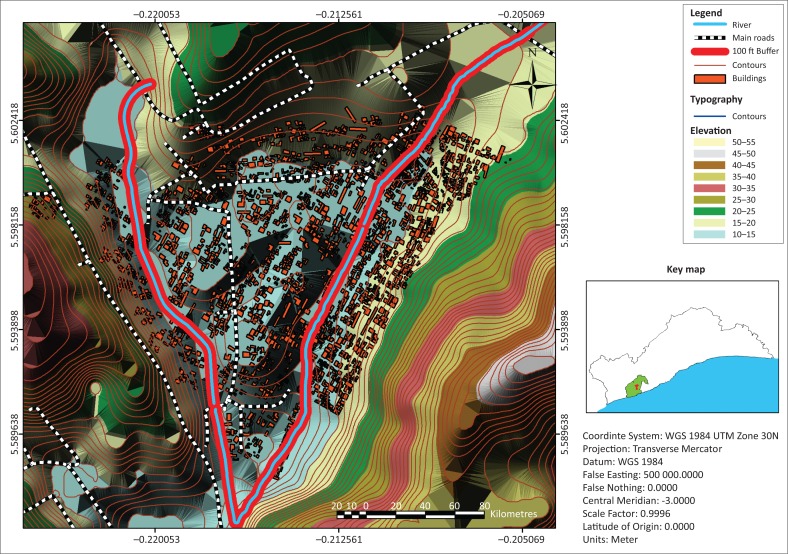
Degree of flood exposure of Alajo using triangular irregular network.

Alajo is one of the rapidly urbanising centres in informal Accra. It is located within the Ayawaso Central sub-metropolitan area of the Accra Metropolis of Ghana (see the land use map of Alajo in Figure 3) and is only 6 km from the Central Business District (CBD) of Accra. The physical area of the community is relatively small, approximately 1 km^2^. The main access road to the community is the Alajo road, a second-class road, which has been encroached by small business activities, and jammed by vehicular traffic and pedestrians’ movements to the CBD. There are small open spaces for recreational activities, and the entire community relies on a school-yard popularly called the ‘polo park’ as a place for social gathering and engagements. The Accra Metropolitan Assembly (AMA) classifies Alajo as a third-class income zone (AMA Medium Term Development Plan 2014), which is the least for such classification, obviously suggesting that poverty levels are really high and there are no indications that the residents can afford rent in formal Accra. The land in Alajo is less than 50 m above sea level, which exposes it to inundations. The community is also located at the confluence of two main rivers, the Odaw River and one of its tributaries, Onyasia River, which easily spread out when there are heavy downpours (refer to Figures 4 and 5). The Odaw River has a watershed of about 200 km^2^ and Alajo is located towards the downstream extent, which is about five miles upstream from Korle lagoon, the point where the Odaw River empties into the Gulf of Guinea. Residents who are closer to the rivers (100–150 feet) are within the flood risk zone identified by the UN-Habitat (2011) which shows the exposure of households to flood (Figures 4 and 5). Impliedly, these people are very liable to floods, but have developed coping strategies to manage and adapt to flood events.

### Materials and methods

This study adopted the convergent parallel mixed method of case study design. This design as indicated by Yin (2003) is flexible and relevant when a detailed and concentrated analysis of a single case is selected for a research. The design also allows data gathering from several sources including interviews, questionnaire administration and observation. The convergent parallel mixed method allows a more thorough understanding of problems through comparisons of diverse perspectives drawn from quantitative and qualitative data (QUAN + QUAL) (Morse 2003). Again, this method of case study design was used as it allows in understanding results and changes needed for a marginalised group by merging their inputs and allowing their perspectives to stand out in a research (Hollohan & Barry 2014). The convergent parallel mixed method can be embraced in a research by designing both quantitative and qualitative strands of research and analysing them in a concurrent manner (Creswell 2012, 2014; Wittink, Barg & Gallo 2006). This has been done, but more emphasis has been placed on qualitative data in relation to quantitative data as an attempt to throw more light on the personal revelation of study participants (as suggested by Hollohan & Barry 2014).

The community-level focus, choice of Alajo, is seen as an appropriate case for study because of its flood history and marginability; thus, we have explored the linkages between the various facets of its informality (housing conditions, livelihoods, legal status and rights), its flood vulnerability and coping measures. Data were extracted from both secondary and primary sources. A blend of both sources gave different accounts of already existing studies for comprehensive analysis. Secondary data sources were reports of preceding research studies, articles and journal papers and government publications. On the other hand, primary data were obtained through field study using interview guides, questionnaires and direct observation. Collection of data through questionnaires took the form of typed and printed predetermined set of questions which were administered to respondents to determine their perspectives on issues related to the subject matter. This was performed by the lead author of the research during 2015–2016 as part of his bachelor’s thesis, who discussed with the respondents a wide range of issues covering informality and floods (including their choice of the community, the economic activities they engage in, number of years and experiences with flood, how they have been able to cope with flood and suggestions they have for flood management).

Alajo lies between two major water bodies (Odaw and Onyasia rivers) which are recognised as the major cause of perennial flood in the community (see Attipoe 2015; Atuguba & Amuzu 2006); hence, households that are within and beyond 100 ft from the rivers were purposively selected. This selection is underpinned by the fact that properties including buildings are not expected to be developed within 100 ft away from the rivers (Town and Country Planning Department 2010). Again, for the purpose of ascertaining the required data, households living in houses as well as shacks and kiosks that are along the rivers were considered. This is to establish the fact that Alajo has some areas which are not flood-prone, and not everyone in Alajo is also poor. The aforementioned criteria served as the basis for inclusion and exclusion of households. Households that qualified based on the criteria were considered, but data were collected from a conveniently selected sample of 60 households. The settlement patterns in the selected parts of Alajo were scattered; thus, the use of probability sampling (systematic and/or simple random sampling) will have taken longer time and efforts as well as greater financial commitment for the research which was based on personal funding. As a result, convenient sampling (opportunity sampling) became a better option in economic sense to enrol households that were ready to participate in the study. As argued by Dörnyei (2007), convenient sampling can be used where conditions surrounding a research are unfavourable for probability sampling to be used. In this case, convenient sampling provides the avenue for researchers to engage field participants who are ready to provide their rich inputs deemed commensurate for drawing valid inferences and making solid analysis. In every house or structure visited, household heads were preferred; however, where household heads were unavailable, data were solicited from household members deemed legitimate to provide data to inform the research.

Apart from households, primary data were solicited from relevant institutions and stakeholders purposively selected, including the National Disaster Management Organisation (NADMO), the AMA and the Town and Country Planning Department (TCPD). Two officers from each institution were selected and interviewed by the lead author. This summed up to six state officials. The total number of field respondents 66, which comprised 60 household heads or representatives and six state officials. The data from primary respondents were supplemented with direct observational technique embraced by the researchers. The drainage patterns were studied to know the direction of flows and their effects on households and coordinates were noted for derivation of contours in the study area. Even though the observations did not follow any organised procedure, they presented various key and relevant issues that were connected to achieve the objectives of the research. Data analysis was performed both qualitatively and quantitatively. The Statistical Package for the Social Sciences (SPSS) software was used to assist in the analysis of the quantitative data, while qualitative data were descriptively analysed.

## Results and discussions

### Major effects of the perennial flood events on the urban residents

The burgeoning studies on urban informality reveal the interconnection between informality and disasters and its associated effects on informal residents (see, for instance, studies such as Elgin & Oztunali 2012; Satterthwaite 2007; Twumasi-Ankrah 1995). Haphazard physical development characterised by concentration and densification of businesses is a common hallmark of informality (Douglas et al. 2008; Fekade 2000). Alajo depicts such features, making the occurrence of floods very common. As a result, the occurrence of floods has produced some negative effects on households (Table 1). The most significant effect has been the loss of household items. This was confirmed by majority of the selected households (68.3%). The disruption and loss of services and businesses by the informal dwellers was confirmed as another effect of the perennial flood. Not only that households confirmed the outbreak of diseases in the community as one outcome of the perennial flood, but also these aforementioned factors were separately confirmed by 11.7% of the households. Other major effects included injuries (3.3%), collapse of buildings (3.3%) and loss of livestock or income-generating activities (1.7%). The flood effects could have direct and indirect implications for the affected people (Jha et al. 2012), which can affect the physical, natural, social and economic well-being of affected members (Douglas et al. 2008). It seems to be true as the flood hazards have significantly influenced the lifestyle and economic conditions of the people. As ‘heroic entrepreneurship’ (as indicated by De Soto in his discussion on informality), Alajo serves as an economic space where individuals conduct their businesses, such as vendors of vegetables, fruits and other food items, cobblers, barbers and second-hand clothes sellers, with many operating in small kiosks who are affected through flood events. These informal workers work without secure contracts and social protection (Debrah 2007); as such occurrences of floods have profound economic implications on them. Again, the quality of life and well-being of households have been affected. This is because Alajo also presents itself as an informal settlement, depicting a true picture of its informality as both economic space and place of abode for people (see Porter et al. 2011; Werna 2001). Because of the occurrence of flood events, many items of affected households such as mattresses, furniture, and so on, become wet, which need to be washed, dried and re-used. This experience is unpleasant, and it takes households’ time away from other important activities, hence reducing their quality of life and well-being. Quite apart from the socio-economic implications of the flood events, households have also been affected psychologically. This is particularly because of insecurity and the continuous effects experienced from the floods, which have translated to the innate local coping strategies to help adapt to floods:

‘Anytime it rains and the gutters are getting full, we begin to panic. We have made our minds to leave here to a safe abode when we are financially ready.’ (Household head, 46 years old, female)

**TABLE 1:** Major effects of preceding floods on residents of Alajo.

Effects	Number of respondents	Percentage
Loss of household items	41	68.3
Outbreak of disease	7	11.7
Disruption and loss of services and businesses	7	11.7
Collapse of building	2	3.3
Personal injuries	2	3.3
Loss of livestock and/or income-generating activity	1	1.7
**Total**	**60**	**100.0**

### Local flood coping strategies

The rise in disasters directly connected to urban informality has negative effects on informal dwellers. This makes informal households to embrace ‘indigenous’ measures to mitigate and respond to flood events. As indicated by Sakijege et al. (2012), informal communities are highly susceptible to flooding but lack support from formal state officials. Informality is closely associated with illegal use of lands for housing purposes, as it does not conform to planning regulations (UN-Habitat 2003), and this makes it difficult for state officials to respond to its needs. In Alajo, for instance, the detrimental effects of flood events characterised by the absence of state support have induced households to adopt ‘indigenous’ means, labelled as ‘coping strategies’ in this article, to mitigate and reactively respond to flooding. These strategies are often short- or long-term efforts taken by individual households that were once victims of flood disaster(s) in order to survive the effects before another one, during or after the event has happened. The households in Alajo predominantly confirmed that despite their threats and insecurity, floods can be coped with when they occur.

One of the key local coping strategies through flood mitigation identified in the community was ‘raised foundations of buildings’, which means that the doors and windows of houses remain at a high level. This approach to cope with flooding has been identified by Sakijege et al. (2012) in a related study in Tanzania. The use of this approach as iterated by households ensures that flood waters cannot easily enter into rooms or inner parts of houses to destroy properties. They further indicated that the physiology of the terrain of Alajo allows for easy flow of water when drains are full, hence making the use of such mitigation strategy a vital one. As victims of previous floods who have gained some considerable experiences, they asserted that the use of this approach has reduced the effects of flooding, especially in relation to the destruction of their properties. In some households, sandbags have been used as a complementary method to block the overflow of water when there are rains. This approach has also been revealed by Adelekan (2010) and Amoako (2012) in their studies of urban informality and floods in Africa. During our field work, one household head (male) had this to say:

‘We have realised that this place will always get flooded during the rainy season. So we decided to raise our buildings to higher heights after some flood encounters which destroyed many people’s properties. This method has helped us other than that, I am sure we will not have been here by now. When it rains and we have floods, the effects are not too massive. Also, some households have used sandbags to block the areas where the water normally flows from. I am very optimistic that next year when it floods, the effects will be very minimal on us.’ (Household head, 37 years old, male)

Apart from raising the foundations of their buildings and using sandbags, the households have embraced proper disposal of wastes and clearing of gutters as a strategic measure to flood hazards. Interactions with households indicated that poor drainage system in the community is a major cause of flood disaster. This coping strategy is usually adopted before and after some days of flood events. With this strategy, the households indicated that they clear their respective drains, especially when the rainy season is approaching. This, according to them, ensures the free flow of water, hence helping in the mitigation of floods. The households further confirmed that in some cases residents who are very close to the two rivers transfer their expensive items to safe places, especially friends and relatives who live nearby. Others pack their items on shelves and other high levels when there are rains. Households also engage in the construction of temporal drains to channel water to appropriate locations, as an attempt to manage flood events. The AMA has not played any role in that regard to support households in their risk mitigation measures. Hence, they are left to fence for themselves. These three aforementioned coping strategies are similar to what Douglas et al. (2008) identified in their study of flooding in Africa. Households added that they have local rescue teams formed, who assist households in the phase of floods as a form of reactive response to floods. The rescue teams entail young men who are locally recognised and contacted by households to help them for an agreed fee. The groups organise themselves in flood-prone areas of Alajo during the rainy season to assist households in quickly transferring their properties to safer places. As stated by a household member:

‘The rescue teams are made up of strong men who help us to move our properties to safe places when we perceive a possibility of flood occurring during the rainy season.’ (Household member, female).

The coping strategies adopted by households have been gauged in terms of their reliability and cost using a Likert scale of ‘high’, ‘medium’ and ‘low’. The choice of scale and the factors considered for the assessment were induced by available data solicited from the study participants. The results are shown in Table 2. The choice of adoption of a particular local coping strategy by households is highly influenced by the financial strength of the households. Hence, while a particular coping strategy could sound very dependable, the cost involved could scare households from embracing it. The majority of the households indicated that they wish they could pull down their buildings and erect new ones with higher foundations, as this seems to work effectively for households that have embraced it. Unfortunately, the cost involved is high; hence, the majority of households prefer to clear their gutters or drains and pack their items on shelves or high levels as these methods are not too expensive.

**TABLE 2:** Average results of reliability and cost of coping strategies: Households’ perspective.

Local coping strategies	Scale for assessment	
	Reliability	Cost
By raising the foundation of building	3	3
By using sandbags to block water flows	2	2
By clearing choked gutters or drains	2	1
By early transfer of items to safe places	2	2
By packing items on shelves and high levels	1	1
By local rescue team	1	2
By constructing temporal drains	2	2

Parameter interpretation: 1, low; 2, medium; 3, high.

### Determinants of local flood coping strategies

Households react to flooding through diverse local coping strategies in order to reduce risks. This is deemed necessary as households living in flood zones will naturally try and reduce the impacts of flooding. However, the intensity of efforts to mitigate and respond to floods could vary depending on differing factors pertaining to a particular flood-prone community. Therefore, we contend that households’ efforts to mitigate and respond to flooding in Alajo are influenced by the following determinant factors of the community which somewhat influence the specific coping strategy or strategies embraced. The determinant factors are the following: (1) the location of Alajo in between two major rivers (locational disadvantage), (2) the limited state support for the flood zones in the community and (3) housing affordability and flexibility of property ownership which entice poor urbanites to stay in the community.

The locational disadvantage of Alajo has been unveiled by Douglas et al. (2008), who indicated that the location of the community automatically makes it a flood-prone area in Accra. This indication goes hand in hand with Satterthwaite (2007), who revealed that informality can contribute to flooding when lower income groups settle on hazardous sites. We support this position as the visits to the community confirmed that it is located between Odaw and Onyasia rivers (a hazardous site for flood occurrence). The households indicated that these rivers overflow their banks when there are heavy rains. Hence, households located very close to the rivers are influenced to deliberately raise the foundations of their buildings as the main coping strategy to deal with flooding. Households that are a bit distant from the river prefer to use sandbags and other materials to block the paths which have been previously identified to be the channels in which the rivers overflow to those households. Thus, the exact locations of households determine which intensive coping strategy they prefer to mitigate and respond to flooding.

‘Because I am too close to the Odaw River, I decided to allow my building to have high foundation so that when there are overflows, it does not destroy my properties.’ (Household head, male)

‘My husband organised other households in the house to raise the blockage you find at the edge of the river. They loaded sand in sacks and bags as well as stones to block the path in which the overflow waters travel.’ (Household member, female)

Discussions with households revealed that city authorities have failed to provide the necessary support and resources to support the community for managing and adapting to floods. They indicated that the state has not supported them in the provision and maintenance of drain infrastructure that contributes to risk reduction. Thus, building, maintaining and clearing drains have been the sole responsibility of the informal urbanites with no state support. According to them, concerned state officials including those responsible for water and sanitation and disaster management have not assisted them in flood risks reduction. For instance, they indicated that the NADMO, responsible for community-based preventive and management education, has failed to educate them on how best they can prevent and manage floods. The lack of formal state support has induced households to put in place informal coping strategies based on their own experiences to deal with the situation. The NADMO officials confirmed that they are challenged with human, financial and logistical resources to effectively operate to support communities to prevent disasters. Discussions with officials from the AMA, a decentralised government body for local level development, confirmed that the government only provides support in the event of floods (reactive response), but the support measures are inadequate because of limited funds. Hence, after flood disasters, relief items are normally provided by some private organisations such as Vodafone Ghana Limited and other philanthropic groups. The ‘hand-outs’ from these non-state bodies are not enough, and also it is not a precise way of dealing with floods. Households prefer precise and sustainable measures which will safeguard their safety. Government, as revealed by the NADMO, is not well positioned to face the full financial burden of relocating community members from Alajo. Again, apart from eviction being a cruel means to deal with the flood, it will not be appropriate as some informal dwellers will later on find their way back to the community (reiterated by a city planner at TCPD). With government not too sure about how to deal with the flood events, households in Alajo are determined to use their little resources to cope differently with flooding in tandem with their divergent experiences with flood events in the community.

In their study, Osei and Ampratwum (2014) identified housing affordability and flexibility of property ownership in informal Accra as a major reason why people prefer to stay in such locations. Our fieldwork confirmed that rent and property costs in Alajo require very low financial resources. Average rent, for instance, is 35.00 Ghana cedis per month as compared to formal Accra where average rent is at a minimum of 120.00 Ghana cedis. Apart from the low cost of living in Alajo, the community is strategically located closer to the (CBD) of Accra, which presents a pool of market for business activities (a hub with concentration of informal economic activities). The majority of households in Alajo engage in informal activities at the CBD. These household members cannot afford the high cost of rent and/or shelter in the CBD and as a result prefer to stay in Alajo which is about 6 km from the CBD. Some households also indicated that they have made investment in acquiring land and other properties in Alajo, and as such find it difficult to abandon them. Others also confirmed that they have their businesses and sources of livelihood at the community and are not financially ready to move to a safer community despite threat. Although they claimed that they will move once they are financially prepared, there are no indications to confirm to the move at any moment from now. Some households live in improvised buildings such as kiosks, containers and uncompleted buildings that lack basic water and sanitation facilities such as potable drinking water and toilet facilities. Satterthwaite et al. (2011) identified that the urban poor live in slums and other informal areas because of housing deficit and the high cost of rent in the cities – a revelation that certainly holds true for Alajo, a slum community. With these compelling factors influencing their stay, the households have been induced to embrace local coping strategies to face flood, which is the major threat to their stay in the community.

### Examining the coping strategies and their contributions to the adaptive resilience of households using the disaster resilience of place model

We start by looking at the informal nature of Alajo in terms of its social systems, natural systems and the physical or built environment, and how they have influenced the degree of inherent vulnerability and inherent resilience of households to flood. Taking into account the social systems, Alajo is a cosmopolitan community, composed of mainly Ga (indigenes), Mole-Dagbons (migrants from the north) and Ewes and Akans (migrants from the south) as ethnic groups (Atuguba & Amuzu 2006). The community has high poverty levels, and its population persistently keeps growing because of its strategic location. The coping measures to flood in the community are mainly household-based instead of entire community’s initiatives. Generally, the physical development of the community is haphazardly organised. Individual households do not coordinate in their efforts to manage flood events; instead, they examine specific situations of their physical environment and find appropriate coping strategies that will help them to specifically manage and adapt to the events. For instance, we realised that households living closer to the two rivers have raised foundations of their buildings, while those closer to major drains clear the drains to allow easy flow of water, especially during rainy seasons. The lack of local coordination in coping with flood contributes to very little success in local flood adaptation in informal communities (Amoako 2012), and this seems to hold true for Alajo. Although the natural systems (in terms of nature of land and location) directly expose the community to flood (as seen as a feature of urban informality as presented by Elgin & Oztunali 2012; Satterthwaite et al. 2011), households have failed to team up to build a strong social system for a supportive physical or built environment which can help them to adapt successfully to flood and subsequently build their resilience to flood over time.

Inferring from the DROP model, the community can be said to have high degree of inherent vulnerability and low degree of inherent resilience, as antecedent conditions of social systems, natural systems and built environment are unfavourable. The immediate effects of preceding floods (loss of household items, disruption and loss of services and businesses, personal injuries, etc.) are influenced by the flood event characteristics (e.g. duration, frequency, intensity, magnitude and rate of its onset). The flood effects can be reduced through coping strategies (Cutter et al. 2008). The coping strategies of households (raising foundations of buildings, using sandbags to block water flows, clearing choked drains, early transfer of items to safer places, packing of items on shelves and high levels, engaging local rescue teams and construction of temporal drains) have yielded some positive results as confirmed by the households. This is a positive indication (+) and has direct positive outcome on the absorptive capacity of households as well as their resilience. Over the years, households have implemented lessons learnt from preceding floods and this has underpinned their differing coping strategies. However, social learning in flood events has been absent, and this makes the absorptive capacity of Alajo very weak. Households revealed that the coping strategies embraced by them are different from one another depending on the specific locations (either closer to the two major rivers, a bit distant from the rivers or at areas closer to major drains) in the community. Households tend to implement coping measures without teaming up with other households, especially those in different houses. Hence, there are no coordination of efforts, knowledge sharing and resource mobilisation to holistically tackle peculiar issues which could be handled by households to deal with floods. This clearly shows the absence of social learning in interventions put in place. Lessons are learnt at individual household level through their own experiences with preceding floods, but these experiences are not shared among affected households to inform sound coping measures needed for strong adaptive capacity for high adaptive resilience to both minor and major flooding. As stated by a household head:

‘Here in Alajo, we all have our own way of dealing with flooding. I have never seen any special gathering instituted by our leaders for us to share ideas on how best to deal with the situation. Members of every house cope with the situation through their own understanding and means which they believe will be enough to mitigate or respond to the event.’ (Household head, male)

**FIGURE 6:**
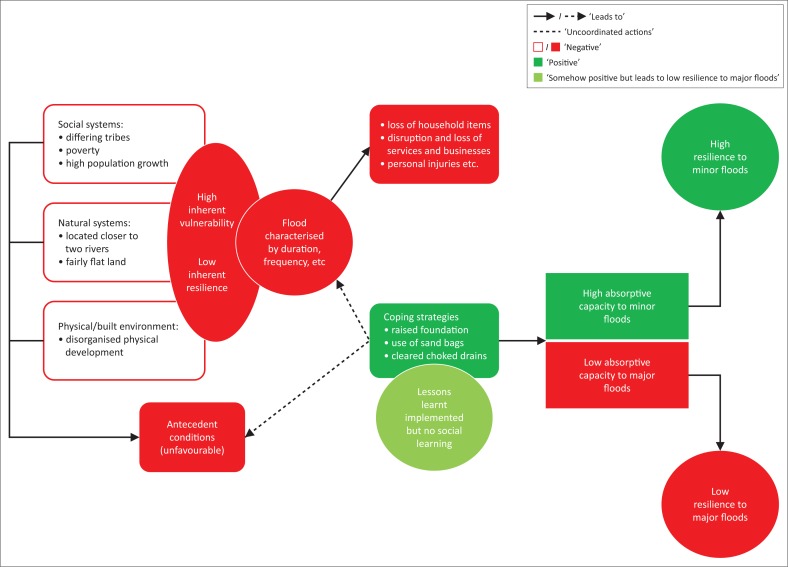
Conceptualised disaster resilience of place model for Alajo.

Adger et al. (2005) have indicated that social learning in community-based disaster prevention and/or mitigation is central and should be embraced by all responsible bodies and individuals in order to tackle disaster events and their detrimental outcomes. They presented that social learning depends on the ability of households to embrace and build strong social integration which allows for group-based actions to deal with a disaster such as flood. This subsequently feeds into the absorptive capacity of households making them more resilient to flood. Because of the absence of social learning, we have doubt in the absorptive capacity of households in Alajo, but we admit that the coping strategies of households (informed by lessons learnt but lack social learning) can help them adapt to minor flood events. In such situations, the absorptive capacity of households will not be exceeded, and their degree of recovery will be very high. In the phase of major flood event, the absorptive capacity of the community will be exceeded and the degree of recovery will as such be very low. Thus, Alajo has high adaptive resilience to minor floods with high degree of recovery but low adaptive resilience to major flood with low degree of recovery (see Figure 6).

## Conclusion

### Remarks towards effective flood management in the context of Alajo with implications for effective urban policy design

Households have taken deliberate steps to cope with flooding in Alajo. The coping strategies have yielded some positive outcomes as confirmed by the households. These measures, however, need to be concretised to ensure effective local adaptation leading to a state of resilience. This is particularly relevant as the strategies implemented by households over the past few years have contributed little to building resilience to flooding. Firstly, we recommend households to take a collaborative approach to handling flood issues through knowledge sharing, and coordinated efforts to help them advance the reliability of their local coping strategies. Social learning is thus one key aspect that households should embrace to build on their adaptive capacity to floods. Secondly, we call on the state not to evict the informal urbanites, and instead locate them in their informality and support their ‘indigenous’ strategies for profound outcomes. Obviously, informality has become part of our cities, and as such we do not expect city authorities to outrightly tag it as a problem (as argued by Roy & Al-Sayyad 2004). Thus, city authorities should offer their support which could also include flood mitigation and adaptation training programmes offered to some community members and groups. Such individuals can later become local pioneers and flood control ‘ambassadors’ who will serve as a channel of exchanges between formal state institutions and the informal urbanites. Also, throughout the research, we realised that households’ coping strategies did not consider rainwater harvesting. Therefore, we contend that the adoption of rainwater harvesting by households in Alajo could be very helpful in managing flooding. This is because of the low-lying nature of the community, which allows for run-offs from rains leading to erosion and ultimately flooding. Apart from the recommendations for local flood management, we wish to make theoretical contribution to the DROP model. In as much as the DROP model has been internalised for community-based disaster management, we call for the model’s inculcation of ‘external supports’ as a measure in concretising coping strategies for building local resilience to flood. Apart from ‘social learning’ which tends to increase the adaptive resilience of communities, ‘external supports’ which are commensurate to managing disasters also contribute to the transformation of disaster vulnerable communities to adaptive resilience communities.
